# Physical Exercise Regulates p53 Activity Targeting SCO2 and Increases Mitochondrial COX Biogenesis in Cardiac Muscle with Age

**DOI:** 10.1371/journal.pone.0021140

**Published:** 2011-07-07

**Authors:** Zhengtang Qi, Jie He, Yuhui Su, Qiang He, Jingxia Liu, Lu Yu, Omar Al-Attas, Tajamul Hussain, Shuzhe Ding, Liu Ji, Min Qian

**Affiliations:** 1 Key Laboratory of Adolescent Health Assessment and Exercise Intervention, Ministry of Education, Shanghai, China; 2 College of Physical Education and Health, East China Normal University, Shanghai, China; 3 Institute of Biomedical Sciences, East China Normal University, Shanghai, China; 4 Center of Excellence in Biotechnology Research, King Saud University, Riyadh, Saudi Arabia; Health Canada, Canada

## Abstract

The purpose of this study was to outline the timelines of mitochondrial function, oxidative stress and cytochrome c oxidase complex (COX) biogenesis in cardiac muscle with age, and to evaluate whether and how these age-related changes were attenuated by exercise. ICR/CD-1 mice were treated with pifithrin-μ (PFTμ), sacrificed and studied at different ages; ICR/CD-1 mice at younger or older ages were randomized to endurance treadmill running and sedentary conditions. The results showed that mRNA expression of p53 and its protein levels in mitochondria increased with age in cardiac muscle, accompanied by increased mitochondrial oxidative stress, reduced expression of COX subunits and assembly proteins, and decreased expression of most markers in mitochondrial biogenesis. Most of these age-related changes including p53 activity targeting cytochrome oxidase deficient homolog 2 (SCO2), p53 translocation to mitochondria and COX biogenesis were attenuated by exercise in older mice. PFTμ, an inhibitor blocking p53 translocation to mitochondria, increased COX biogenesis in older mice, but not in young mice. Our data suggest that physical exercise attenuates age-related changes in mitochondrial COX biogenesis and p53 activity targeting SCO2 and mitochondria, and thereby induces antisenescent and protective effects in cardiac muscle.

## Introduction

Cardiac muscle undergoes a significant remodeling with age, and is mostly characterized by a significant loss of cardiac myocytes, reactive hypertrophy of the remaining cells and increased connective tissue. Cardiac muscle also undergoes a hypertrophic remodeling with strength training or regular endurance exercise that is known to increase muscle mass or promote cardiac functional capacity. Age-related cardiac remodeling really contributes to cardiac dysfunctions and even diseases in old animals. Cardiac dysfunction partly results from the progressive impairment of mitochondrial biogenesis and function with age [1]. Mitochondrial reticulum was dynamic, sensitive and plastic in response to oxidative stress [2], mitochondria contributed to cardiac dysfunction and myocyte apoptosis via a loss of metabolic capacity and by the production and release of reactive oxygen species (ROS) [3]. Mitochondrial oxidative stress appears to play a central role in cardiac aging. Cardiac aging in mice is accompanied by accumulation of mitochondrial protein oxidation, increased mitochondrial DNA(mtDNA) mutations and deletions, increased mitochondrial biogenesis, increased ventricular fibrosis, enlarged myocardial fiber size, decreased cardiac sarco(endo)plasmic reticulum Ca^2+^-ATPase(SERCA2) protein, and activation of the calcineurin-nuclear factor of activated T-cell pathway. All of these changes with age are attenuated by overexpressing catalase targeted to mitochondria [4], [5]. The application of mitochondrial antioxidants or nutrients is hoped to delay cardiac aging, prevent ROS-related cardiovascular diseases, diabetes, Alzheimers' disease, immune dysfunction, and prolong median lifespan of people [6], [7], [8].

On the other hand, chronic ROS production and oxidative stress is necessary for cell insulin signaling and mitochondrial biogenesis. Chronic ROS production by mitochondria may contribute to the development of insulin resistance, a primary feature of type 2 diabetes, but mice lacking glutathione peroxidase 1 were protected from high-fat-diet-induced insulin resistance. The studies provide causal evidence for the enhancement of insulin signaling by ROS in vivo [9]. Probably, it is the degree of ROS generation and the context that determines whether ROS enhance or suppress insulin sensitivity. Exercise practitioners often take vitamin C supplements because intense muscular contractile activity can result in oxidative stress, but Vitamin C supplementation prevented the exercise-induced expression of cytochrome C (a marker of mitochondrial content) and of the antioxidant enzymes. The adverse effects of vitamin C may be due to its capacity to reduce the exercise-induced expression of key transcription factors involved in mitochondrial biogenesis [10]. Despite vitamin C prevents increases in skeletal muscle mitochondrial biogenesis and antioxidant enzymes with exercise training, high-dose antioxidant vitamin C supplementation does not prevent acute exercise-induced increases in markers of skeletal muscle mitochondrial biogenesis [11]. These data suggest chronic ROS production and subtle oxidative stress is necessary for mitochondrial biogenesis, it is not always reasonable for aging and ROS- related diseases to decrease ROS production no matter what happens. Transient ROS produced by physiological stimuli such as exercise may be beneficial, sustained mitochondrial ROS generation may be pathogenic.

Cytochrome c oxidase (COX, complex IV) biogenesis is a part of mitochondrial biogenesis. COX consists of thirteen subunits. Of which, the largest subunits, COX I–III, are encoded by the mtDNA and activated in transcriptional level by mitochondrial transcription factors Tfam and TFB2M. TFB1M and TFB2M are necessary for basal transcription of mammalian mitochondrial DNA. Both TFB1M and TFB2M interact directly with mitochondrial RNA polymerase, allowing flexible regulation of mtDNA gene expression in response to the complex physiological demands of mammalian metabolism [12]. The largest subunits constitute the catalytic core of the enzyme; the rest of the smaller subunits are encoded by nDNA and activated in transcriptional level by nuclear transcription factors NRF-1, NRF-2, Sp1, YY1 and MEF2A. Most of these transcription factors are downstream effectors in peroxisome proliferator-activated receptor gamma(PPARγ), estrogen-related receptor alpha(ERRα), PPARγ coactivator alpha(PGC-1α) signaling cascades, members of which are often considered as the markers of mitochondrial biogenesis. The COX biogenesis relies on a number of assembly proteins, including cytochrome oxidase deficient homolog 1, 2 (SCO1, SCO2), COX11, COX15, Surf1p, COX17 and SURF1, which are essential for the correct assembly and stability of this complex. SCO1, SCO2 and COX17 are also responsible for delivery of copper ions to the mitochondrion and for insertion of these ions into the enzyme and maintenance of cell copper homeostasis [13]. Of these assembly proteins, SCO2 is a p53-targeted gene that modulates the balance between mitochondrial respiration and glycolytic pathways [14]. Mutations in SCO2 results in infantile hypertrophic cardioencephalomyopathy and a severe COX deficiency in striated muscle [15]. Structural and histochemical studies indicates that the cardiomyopathic phenotype was associated with compound heterozygosity (E140K with another nonsense mutation) in the SCO2 gene [16]. These intriguing data prompted us to hypothesize that p53-targeted SCO2 and COX biogenesis may be induced by exercise.

Generally, nuclear p53 functions as a transcription factor for target genes regulating apoptosis, cell cycle, cell respiration and metabolism. This mechanism can reason that cancer cells lacking p53 expression due to p53 mutation are inclined to proliferation and glycolytic pathways, and cells and mice overexpressing p53 are prone to apoptosis, senescence and mitochondrial respiration. We hypothesized that the homeostasis of p53 expression may be prolonged by exercise. Alternatively, p53 is also distributed into mitochondria and regulate apoptosis and oxidative stress by a transcription- independent pathway. This cell death did not require the transcription of p53 target genes and was preceded by the translocation of p53 into mitochondria. p53 accumulation in mitochondria was the critical factor for eliciting p53-dependent but transcription-independent apoptosis [17]. p53 translocation to mitochondria precedes its nuclear translocation and targets oxidative defense protein manganese superoxide dismutase (MnSOD), a p53-regulated gene that is a vital antioxidant enzyme localized in mitochondria. In the mitochondria, p53 interacts with MnSOD, consistent with the reduction of its superoxide scavenging activity, and a subsequent decrease of mitochondrial membrane potential [18]. MnSOD deficiency enhances adriamycin and paclitaxel-induced p53 levels in heart mitochondria [19]. Mitochondria can regulate p53 activity, and assaults on the cell that affect mitochondrial ROS production and mitochondrial function can influence p53 activity [20]. In the autophagic process, contrasting with the fact that nuclear p53 induces autophagy through transcriptional effects, the cytoplasmic p53 acts as a master repressor of autophagy in multiple experimental settings [21]. We hypothesized that subcellular distribution of p53 may be changed by exercise, and play a role in exercise-induced cardiac adaptation.

Therefore, the first aim of this study was to outline the timelines of mitochondrial function, oxidative stress and COX biogenesis in cardiac muscle with age. We further investigated whether and how endurance exercise, known to successfully promote cardiac functional capacity, exerts an effect on COX biogenesis. We determined changes in mtDNA content and expression of COX subunits and assembly proteins, and mitochondrial biogenesis markers with endurance exercise. The multiple roles of p53 in ROS homeostasis [22], mitochondrial-dependent apoptosis, and COX assembly and mitochondrial respiration, prompted us to hypothesize that regular exercise would regulate p53 expression and distribution, and maintain the cross-talk homeostasis between mitochondrial ROS and p53 activity in older mice, thereby potentially delaying cardiac aging.

## Results

### 1. Mitochondrial ROS production and oxidative stress

**Figure 1:**
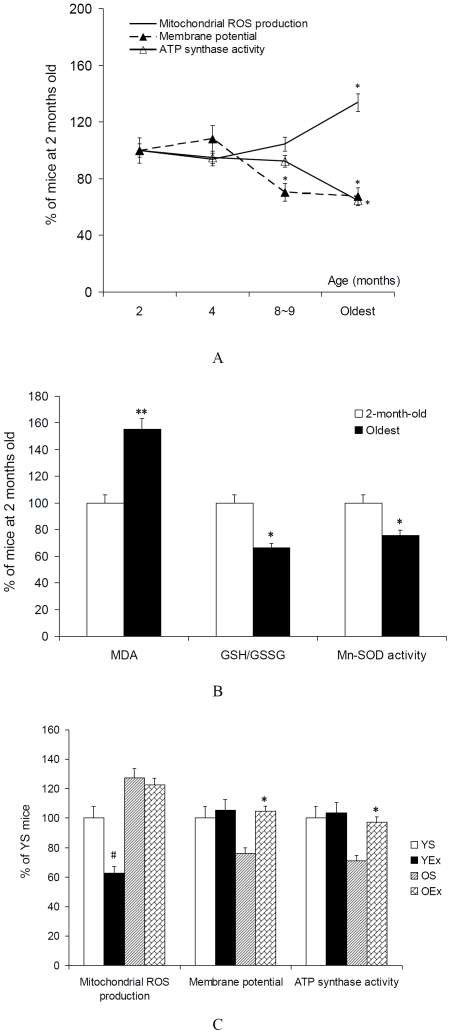
Mitochondrial function and oxidative stress in cardiac muscle. All data are presented as mean ± SEM (n = 7∼9) and displayed as percent of mice at 2 months of age (YS mice). A: Mitochondrial ROS production, membrane potential and ATP synthase activity from mice at different age. The statistical significance of differences was calculated by using one-way ANOVA. *P<0.05, **P<0.01 vs. mice at 2 months of age. B: MDA level, GSH/GSSG and Mn-SOD activity in mitochondrial fractions from mice at different age. The statistical significance of differences was calculated by Student's t-test. *P<0.05, **P<0.01 vs. mice at 2 months of age. C: Mitochondrial ROS production, membrane potential, ATP synthase activity in cardiac muscle from exercised mice. The statistical significance of differences was calculated by students' t test. *P<0.05, **P<0.01 vs. OS mice, #P<0.05 vs. YS mice.

As shown in Fig. 1, ROS production does not increased markedly until the oldest age (P<0.05), exercise training decreased ROS production in young mice (P<0.05), but not in old mice. Mitochondrial membrane potential decreased at 8∼9-month-old and the oldest age (P<0.05), exercise training increased membrane potential in old mice (P<0.05), but not in young mice. MDA level increased (P<0.01), and GSH/GSSG ratio and Mn-SOD activity decreased at the oldest age (P<0.05). As a biomarker of mitochondrial function, ATP synthase activity does not decreased markedly until the oldest age (P<0.05), exercise training increased ATP synthase activity in old mice (P<0.05), but not in young mice.

### 2. mtDNA markers content

**Figure 2:**
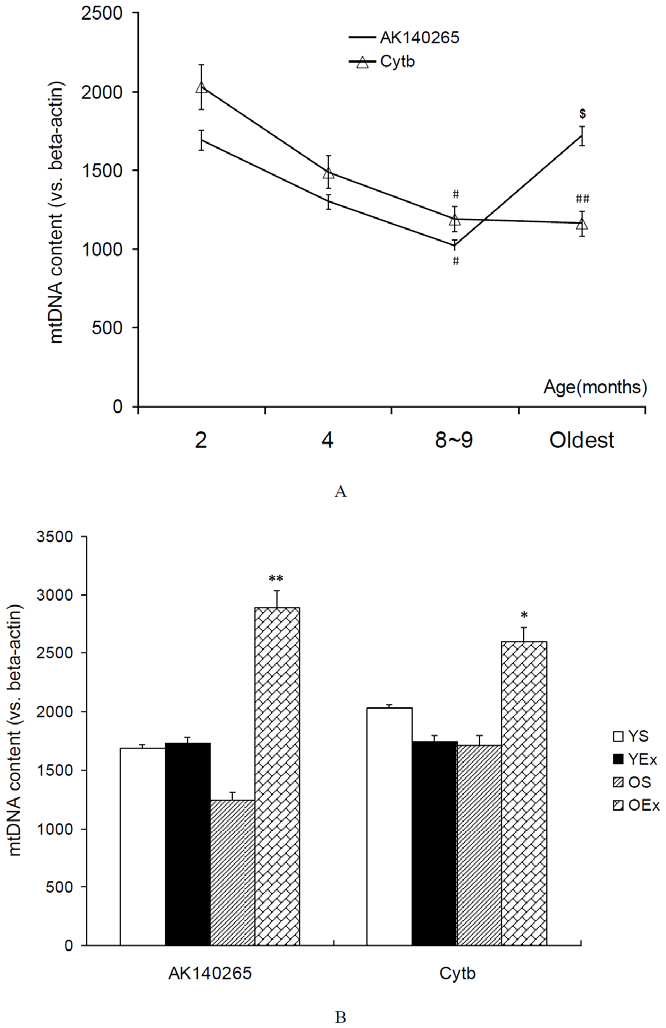
mtDNA markers content in cardiac muscle. All data are presented as mean ± SEM (n = 8) and displayed as arbitrary units relative to β-actin, nuclear DNA marker. A: mtDNA markers content from mice at different age. The statistical significance of differences was calculated by using one-way ANOVA. #P<0.05, ##P<0.01 vs. mice at 2 months of age, $ P<0.05 vs. mice at 6 months of age. B: mtDNA markers content from exercised mice. The statistical significance of differences was calculated by students' t test. *P<0.05, **P<0.01 vs. OS mice.

The replication of mtDNA is a central marker of mitochondrial biogenesis. mtDNA encodes 37 genes including 2 rRNAs, 22 tRNAs, and 13 polypeptides. Of the 13 proteins, cytochrome b(Cytb) is an important part of complex III, ubiquinol-cytochrome-c reductase. AK140265 is an inserted and noncoding sequence located at ChrM:70-852 in adult male mice (http://genome.ucsc.edu). As shown in Fig. 2, the gene copies of Cytb decreased significantly at 8∼9-month-old (P<0.05) and the oldest age (P<0.01), exercise training increased the copies of Cytb in old mice (P<0.05). Unexpectedly, we found that the copy number of AK140265 decreased significantly at 8∼9 months of age (P<0.05 vs. 2-month-old), but was reelevated at the oldest age (P<0.05 vs. 8∼9 -month-old). Exercise training increased the copies of AK140265 in old mice (P<0.01), but not in young mice.

### 3. COX subunits and assembly proteins

**Figure 3:**
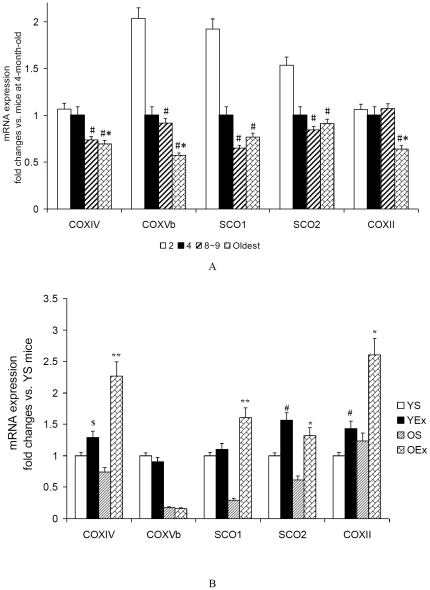
Gene expression of COX subunits and assembly proteins in cardiac muscle. A: Gene expression of COX subunits and assembly proteins from mice at different age. Data are presented as mean ± SEM (n = 8) and displayed as fold changes vs. mice at 4 months of age. The statistical significance of differences was calculated by using one-way ANOVA. *P<0.05 vs. mice at 4 months of age, #P<0.05 vs. mice at 2 months of age. B: Gene expression of COX subunits and assembly proteins from exercised mice. Data are presented as mean ± SEM (n = 8) and displayed as fold changes vs. YS mice. The statistical significance of differences was calculated by students' t test. *P<0.05, **P<0.01 vs. OS mice, #P<0.05, $ P = 0.064 vs. YS mice.

The COX enzyme activity and subunits expression are usually depressed in copper-deficient and hypertrophic cardiac disease [23], [24], [25]. Of the COX subunits, SCO1 and SCO2 are metal Cu chaperones; that are essential for the assembly of the catalytic core of COX. Cu can restore COX activity that is depressed in hypertrophic cardiomyocytes, and COX plays a determinant role in Cu-induced regression of cardiomyocyte hypertrophy [26]. A study suggests that a mitochondrial pathway for the regulation of cellular copper content that involves signaling through SCO1 and SCO2, perhaps by their thiol redox or metal-binding state [27]. We asked whether the induction of oxidative stress by exercise in cardiac muscle is also accompanied by the regulation of genes of COX subunits and assembly factors. As shown in Fig. 3, the increase in age reduced the transcription of several COX subunit genes, including cytochrome c oxidase complex subunit II, IV, Vb(COXII, COXIV, COXVb) and some genes involved in the regulation of COX assembly, including SCO1 and SCO2 (P<0.05). The data bar also showed us that the downregulation of these genes did not emerged at the same age. Exercise training increased the transcription of COXII, COXIV, SCO1 as well as SCO2 (P<0.05), did not affect COXVb expression.

### 4. Transcription cascade involved in mitochondrial biogenesis

**Figure 4:**
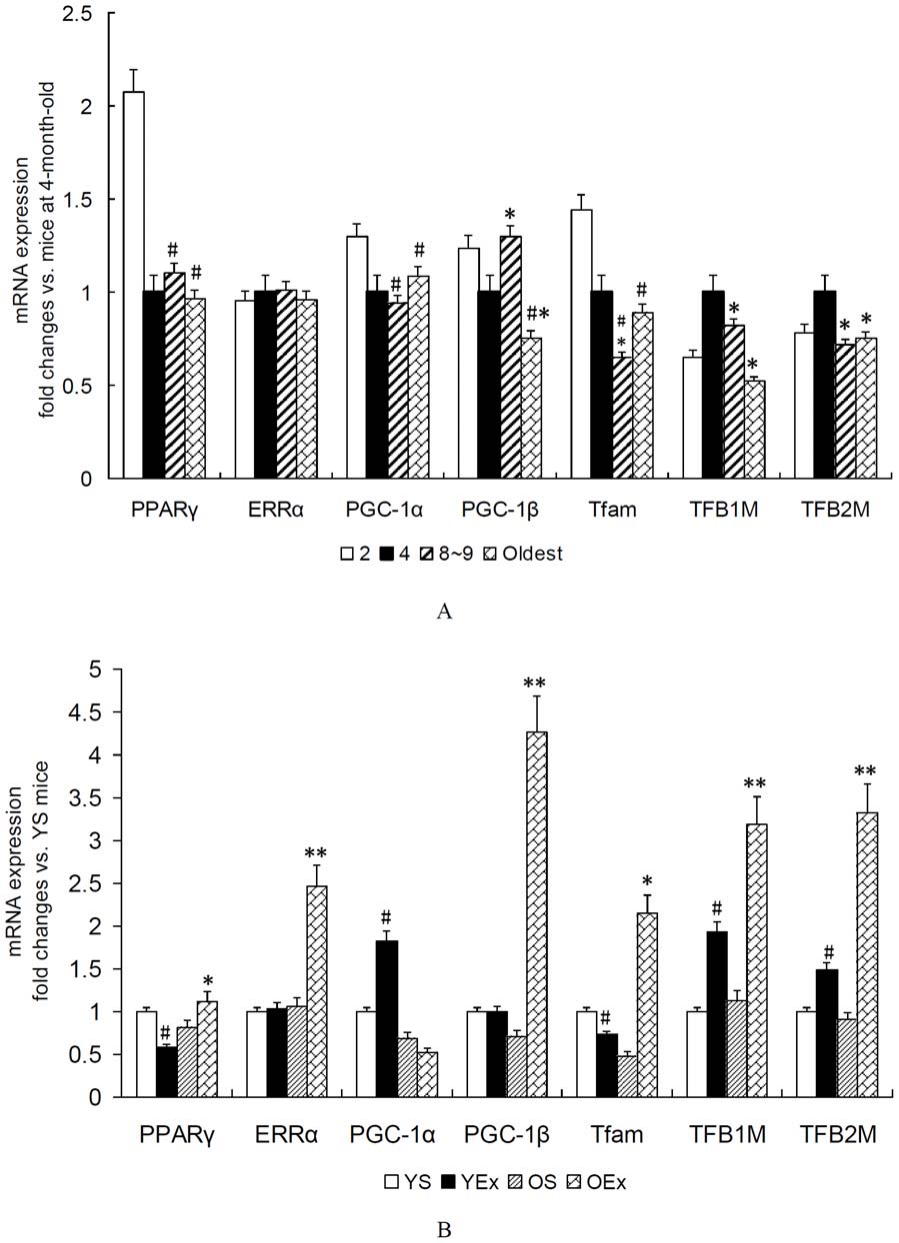
Mitochondrial transcription cascade in cardiac muscle. A: Mitochondrial transcription cascade from mice at different age. Data are presented as mean ± SEM (n = 8) and displayed as fold changes vs. mice at 4 months of age. The statistical significance of differences was calculated by using one-way ANOVA. *P<0.05 vs. mice at 4 months of age, #P<0.05 vs. mice at 2 months of age. B: Mitochondrial transcription cascade from exercised mice. Data are presented as mean ± SEM (n = 8) and displayed as fold changes vs. YS mice. The statistical significance of differences was calculated by students' t test. *P<0.05, **P<0.01 vs. OS mice, #P<0.05 vs. YS mice.

To identify the transcription cascade responsible for the regulation of COX subunits, we focused on the markers of mitochondrial biogenesis, since activation of these transcription factors and coactivators by exercise is known to contribute to mitochondrial biogensis in skeletal muscle [28], [29]. Tfam, TFB1M and TFB2M are essential for mtDNA replication and transcription of COXI, COXII and COXIII [30], the coordinate regulation of nuclear-encoded mitochondrial transcription factors by NRFs and PGC-1 family coactivators is essential to the control of mitochondrial biogenesis [31]. The two initiation factors, Tfam and TFB2M, and two promoters, LSP and HSP1, are required to drive transcription of the mitochondrial genome [32]. As shown in Fig. 4, except ERRα, mitochondrial biogenesis-related genes expression in the transcription cascade were almost depressed in the oldest mice vs. 2∼4 months of age (P<0.05). These reductions in gene expression were not linearly correlated with age. In old mice, exercise training upregulated the expression of genes in the transcription cascade (P<0.05), except PGC-1α. In young mice, these inductions of exercise are also marked in expressions of PGC-1α, TFB1M and TFB2M (P<0.05). We did observed that exercise training depressed the expressions of PPARγ and Tfam in cardiac muscle of young mice (P<0.05).

### 5. p53 expression and p53 protein content in mitochondria

**Figure 5:**
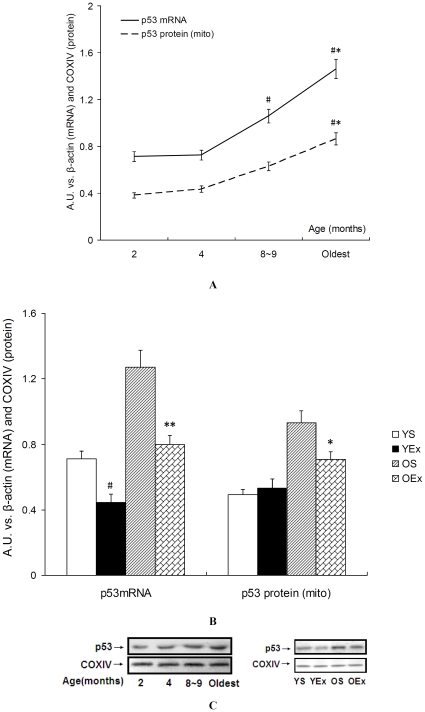
p53 mRNA expression and p53 protein content in mitochondria. Data are presented as mean ± SEM and displayed as arbitrary unit (A.U.) vs. β-actin (mRNA, n = 8) and COXIV (protein, n = 4). A, C: Effects of age on p53 mRNA level and protein content in mitochondria. The statistical significance of differences was calculated by using one-way ANOVA. *P<0.05 vs. mice at 4 months of age, #P<0.05 vs. mice at 2 months of age. B, C: Exercise decreased p53 mRNA level and p53 translocation into mitochondria in older mice. The statistical significance of differences was calculated by students' t test. *P<0.05, **P<0.01 vs. OS mice, #P<0.05 vs. YS mice.

As a pivotal transcription factor, p53 is not considered as a biomarker of mitochondrial biogenesis in transcription cascade. However, the fact that p53 regulates mitochondrial respiration by SCO2 and TIGAR makes it convinced that p53 is essential for mtDNA maintainance and replication [33], [34], and has a direct positive effect on mitochondrial biogenesis and function [35], [36]. p53 has an exciting role in maintaining mitochondrial genetic stability through its ability to translocate to mitochondria and interact with mtDNA polymerase gamma (pol gamma) in response to mtDNA damage induced by exogenous and endogenous insults including ROS [37]. We therefore asked whether the responses of oxidative stress and mitochondrial biogenesis to exercise in cardiac muscle is also accompanied by the regulation of p53 expression and mitochondrial translocation. As shown in Fig. 5, expression level for p53 gene increased significantly at 8∼9-month-old and the oldest age (P<0.05, Fig. 5A), exercise training significantly lowered expression level for p53 as compared to control mice respectively (P<0.05, Fig. 5B). Western blot analysis confirmed that protein level for p53 in mitochondrial fractions was higher in the oldest heart muscle as compared to 2∼4 months of age (P<0.05, Fig. 5A, C). Exercise training decreased protein level for p53 in old mice (P<0.05, Fig. 5B, C), but not in young mice.

### 6. p53 activity as a transcription factor targeting SCO2

**Figure 6:**
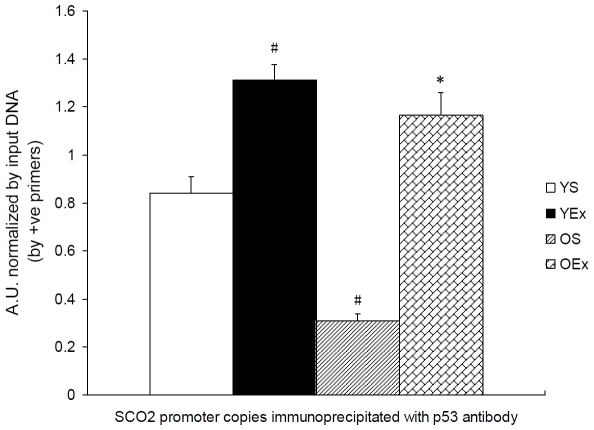
Exercise altered the SCO2 promoter copies binded with p53 antibody. Data are presented as mean ± SEM (n = 7) and displayed as arbitrary unit (A.U.) vs. input DNA. The SCO2 promoter copies were detected by real-time PCR using +ve primers. The statistical significance of differences was calculated by students' t test. *P<0.05, vs. OS mice, #P<0.05 vs. YS mice.

Aging and exercise-induced adaptation have been associated with widespread changes at the gene expression level in multiple mammalian tissues. Age-related induction of p53-related genes was observed in multiple tissues, but oxidative stress does not induce the expression of these genes. These observations support a role for p53-mediated transcriptional program in mammalian aging and suggest that mechanisms other than ROS are involved in the age-related transcriptional activation of p53 targets [38]. To identify the role of p53 as a transcription factor targeting SCO2 in COX biogenesis during exercise, Chromatin immunoprecipitation(ChIP) assays was used to quantify the SCO2 promoter copies immunoprecipitated with p53 antibody(Table S2, Fig.S1). In agreement with the above data that the increase in age depressed SCO2 expression since 8∼9 months old, we observed that the p53 binding to the SCO2 promoter was higher in young mice as compared to the old mice (P<0.05, Fig. 6).We also observed that exercise training increased the p53 binding to the SCO2 promoter in young and old mice (P<0.05, Fig. 6).

### 7. Blocking p53 mitochondrial translocation increases mitochondrial COX biogenesis in old mice

**Figure 7:**
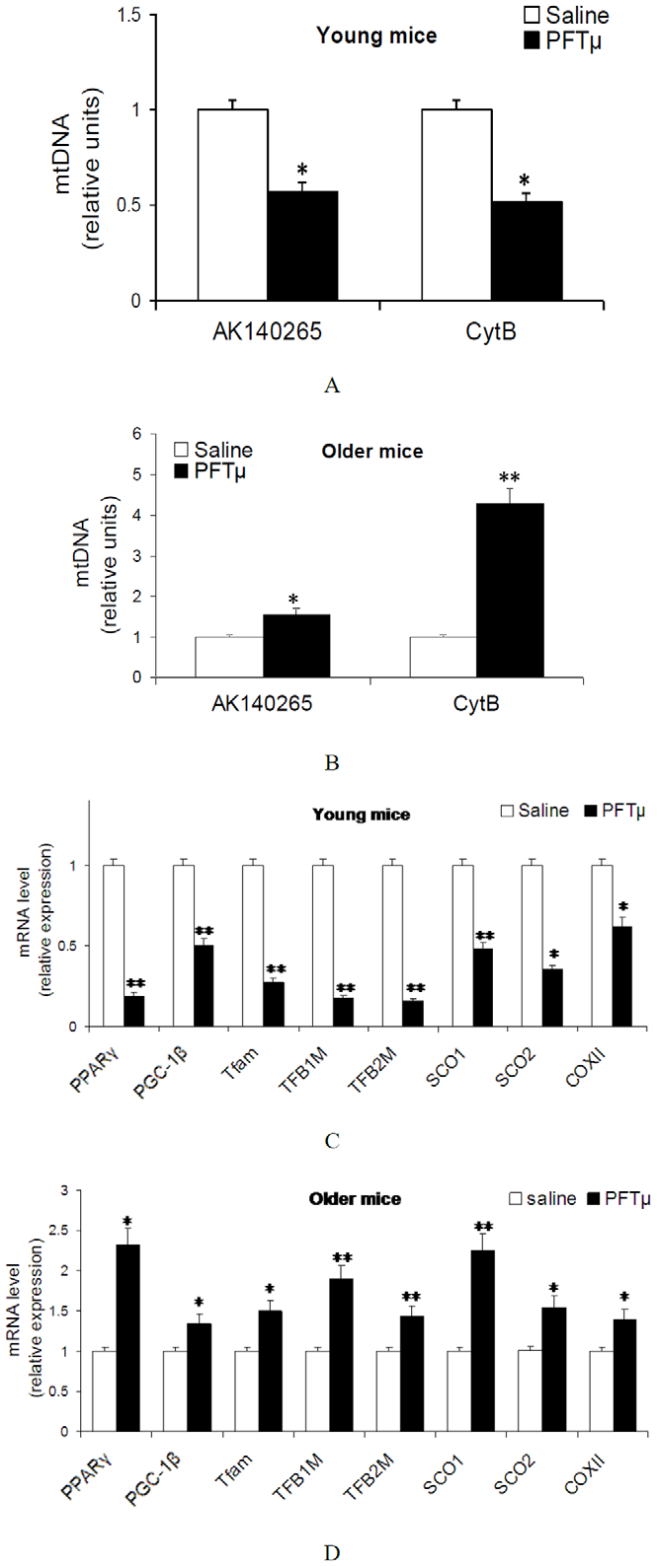
Blocking p53 mitochondrial translocation increases mitochondrial COX biogenesis in the older mice (10∼11-month-old). A, B: mtDNA content. C, D: Gene expression of COX subunits and assembly proteins, and the transcription cascade involved in mitochondrial biogenesis. Data are presented as mean ± SEM (n = 8) and displayed as fold changes vs. control mice (treated with saline). The statistical significance of differences was calculated by students' t test. *P<0.05, **P<0.01 vs. Saline.

To identify the role of p53 mitochondrial translocation on mitochondrial COX biogenesis, and support the effects of exercise, pifithrin-μ was used as an inhibitor of p53 binding to mitochondria. Because pifithrin-μ shuts down only the p53-mitochondrial pathway without affecting the transcriptional functions of p53, it is superior to pifithrin-α. As shown in Fig. 7, we found that PFTμ increased mtDNA content in older mice, but not in young mice (P<0.05, Fig. 7A, B). Meanwhile, PFTμ increased the expression of genes including mitochondrial biogenesis markers, COX subunits and assembly proteins in older mice, but not in young mice (P<0.05, Fig. 7C, D).

## Discussion

The main objective of this study was to find a correlative evidence that exercise would regulate oxidative stress and COX biogenesis via p53 activity targeting SCO2 and mitochondria, based on the fundamental role of p53 in ROS homeostasis and mitochondrial biogenesis. This study has generated three additional lines of information regarding the effects of exercise on p53 expression and COX biogenesis in cardiac muscle, and proposed a novel correlation that p53 activity may be implicated in exercise-induced cardiac COX biogenesis.

First, we demonstrated that mRNA expression of p53 and its protein levels in mitochondria increased with age, accompanied by increased mitochondrial oxidative stress, reduced expression of COX subunits and assembly proteins, and decreased expression of most markers in mitochondrial transcription cascade of mice cardiac muscle. Mitochondrial dysfunction and oxidative stress contribute to cardiac aging, a decline in mitochondrial function coupled with the accumulation of oxidative damage to mitochondria may be causal to the decline in cardiac performance with age [5], [39], [40], [41]. The results presented here showed that the decline in mitochondrial membrane potential(Δψ) was marked in heart muscle of mice at 8∼9 months of age, when ROS production was not increased significantly and ATP synthase activity was not impaired. Until the oldest age, we observed that cardiac oxidative stress increased in all markers and resulted in the impaired mitochondrial function. Our data confirmed that mitochondrial dysfunction and oxidative stress were involved in the early phase of cardiac aging that appeared to be a progressive process, with an asynchronous change of oxidative stress markers during the age frame shown here.

Our findings at the oldest age showed that the increase in age decreased the expression of mtDNA-encoded COXII, nDNA-encoded COXIV and COXVb, and PPARγ/PGC-1α, β transcriptional cascade. COX is a specific intra-mitochondrial site of age-related deterioration. A study on drosophila showed that COX activity declined progressively with age by 33%, western blot analysis showed that the abundance of the COX subunits IV, Va, Vb, VIb, VIc, VIIc, and VIII decreased ranging from 11% to 40% during aging [42]. Upon synaptic mitochondria, COX activity is currently considered an endogenous marker of neuronal oxidative metabolism; changes of mitochondrial ultrastructure and metabolic competence may contribute to the age-related alterations of neuronal performances [43]. The levels of COXIV and COXII proteins in the cerebellar Purkinje cells were reduced in age-matched controls relative to young controls, and in the Alzheimer's disease group relative to both age-matched and young controls. Results suggest that irregular distribution of COX subunits is causal to aging and Alzheimer's disease [44]. Our data supported that impaired COX biogenesis also occurred in the heart of older mice, which maybe had an adverse impact on mitochondrial physiology during the early phase of aging. A challenging study found an increased mitochondrial and nuclear gene expression of COX subunits I and IV in neuronal aging, the findings sustained that gene expression of COX subunits I and IV was not to be involved in the well-documented time- related mitochondrial decay [45]. Furthermore, age-related reductions of mtDNA copy number occurred in skeletal muscle and liver, but not in the heart. mtDNA levels are preserved in the aging heart muscle, presumably due to its incessant aerobic activity [46]. Our data disagreed with the author's findings and proposed a paradox. The content of mtDNA marker Cytb was shown to decline with age. Surprisingly, the copy number of AK140265, an inserted and noncoding sequence, was initially decreased and then increased during the age frame shown here. These findings suggest that the copy number of one sequence of mtDNA cannot really indicate the copy number of mtDNA. We surmise that the accumulation of mtDNA damages during aging contains not only the decreased copy number of mtDNA encoding sequences, but also the increased copy number of mutated, inserted and noncoding sequences. Taken together, it is reasonable to suppose that any alteration of the copy number of mtDNA- encoded gene, gene expression of COX subunits and assembly proteins reported to occur in cardiac muscle of old mice may significantly affect the proper assembling of the COX and its activity. The proper functioning of COX depends on several factors that can affect mitochondrial metabolic competence in the aging cell. Mitochondrial COX function relies on the overall balance among various determinants that can be differently damaged by aging and represent causative events responsible for the age-related functional decline of selected mitochondrial populations [45]. Therefore, the present study provides an evidence that COX biogenesis has been impaired in the early phase of cardiac aging via PPARγ/PGC-1α, β transcriptional cascade, known to be the most important pathway to mitochondrial biogenesis.

Second, our data in older mice demonstrated that endurance exercise decreased mRNA expression of p53 and its protein levels in mitochondria of cardiac muscle, accompanied by changes in expression of COX subunits and assembly proteins, and markers in mitochondrial transcription cascade. The data presented here showed that exercise increased mitochondrial Δψ and ATP synthase activity in older mice, but did not affected ROS production. Hearts of old mice contain a subpopulation of myocytes with reduced mitochondrial stress-tolerance that is attributed to an age-dependent reduction of cellular ROS defence capacity [47]. Our data suggest that, to a certain extent, endurance exercise can attenuate age-related changes in cardiac mitochondria, thus to maintain their capacity to produce ATP. The fact that exercise increased mitochondrial content and biogenesis in skeletal muscle was repeatedly confirmed [28], [48], [49], [50], [51], [52], [53]. PPARγ/PGC-1α, β transcriptional cascade plays a central role in mtDNA replication and transcription [29], [30]. However, little attention was focused on mitochondrial biogenesis in cardiac muscle, presumably due to the higher distribution of mitochondria in cardiac myocyte and the lower sensitivity in response to exercise. It is inferred that chronic exercise exerts a whole-body beneficial effect that exceeds muscle adaptation, likely through mechanosensitive afferent nerves and beta-endorphin release to brain and plasma that promote mitochondrial biogenesis in distant organs [54]. This inference prompts us to hypothesize that mitochondrial biogenesis in distant organs during or after exercise may be promoted through endocrine and intercellular signaling, instead of mechanical stimuli on cells. To identify whether exercise promoted COX biogenesis in cardiac muscle, thus to improve cardiac function, our results clearly demonstrated that, in older mice, exercise increased mtDNA content and expression of COX subunits and assembly proteins, and mitochondrial biogenesis markers. It was only expression of COXVb and PGC-1α that was unchanged. There is a pattern of increasing mitochondrial and nuclear gene expression for OXPHOS enzymes in developing cardiac tissue and decreasing OXPHOS gene expression in the aging heart [55]. We concluded here that, at least to a certain degree, exercise could increase cardiac COX biogenesis and prevent the decreasing COX gene expression in the older mice. The mRNA levels of some COX genes were increased 2.0∼5.0 folds by exercise, and even surpassed their mRNA levels at young age. In response to exercise, the mRNA expression of these genes of older mice showed more responsive than that of younger animals.

Third and the most important, p53 expression and its activity targeting SCO2 transcription and mitochondria were evaluated in this study. p53 expression and its activity play a double-edged role in cell metabolism, apoptosis and aging. Lack of p53 causes inhibited mitochondrial respiration, reduced mtDNA maintenance and increased oxidative stress [14], [33], [34]. It has been revealed that p53 regulates energy metabolism, oxidative stress, and amino acid metabolism through balancing glycolysis and oxidative phosphorylation (OXPHOS) as well as the autophagy pathway. p53 is activated by metabolic stress through AMPK and mTOR signaling pathways. p53 regulates OXPHOS through a p53 response element in the promoter of the gene of SCO2, an important protein directing the assembly of the COX complex of the electron transport chain [14], [56], [57]. ChIP assays presented here that the increase in age reduced the SCO2 promoter copies binded with p53 in cardiac muscle, and exercise increased the p53 binding to the SCO2 promoter in young and old mice. Meanwhile we also found that the increase in age reduced the SCO2 mRNA level, and exercise increased SCO2 expression in young and old mice. These results lead us to a conclusion that p53 activity targeting SCO2 transcription plays a role in age-related cardiac mitochondrial COX decay and effects of exercise.

P53 can also affect ROS production in mitochondria and balance cellular oxidative stress. p53-null cells exhibit a disruption of cellular ROS homeostasis and mtDNA maintenance, characterized by mtDNA depletion, reduced mitochondrial and cellular superoxide levels and increased cellular hydrogen peroxide [34]. Mitochondrial antioxidant enzyme MnSOD is regulated by p53 through transcription-dependent pathway, and this regulation is important for controlling ROS production in mitochondria. In turn, mitochondrial ROS affect p53 transcriptional activity and subcellular loaction [20]. Cellular generation of ROS is central to redox signaling, p53 is a redox-regulated transcription factor that binds specifically to DNA and activates transcription of target genes, including SCO2 and MnSOD. In addition to being a transcription factor, p53 can regulate oxidative stress and apoptosis through transcription-independent pathway. p53 directly targets mitochondria to decrease mitochondrial Δψ and increase apoptosis [58], [59], [60]. p53 interacts with MnSOD in mitochondria, and this interaction correlates with diminished MnSOD enzyme activity [18]. Consequently, p53 activity targeting mitochondria may increase mitochondrial ROS production and oxidative stress. In turn, mitochondrial-generated ROS may also induce p53 translocation to mitochondria and stimulate mitochondrial oxidative stress, leading to an increase in apoptosis. Recent studies have revealed that cellular concentration and distribution of p53 has a distinct cellular function, and that ROS act as both an upstream signal that triggers p53 activation and a downstream factor that mediates apoptosis [22]. There may be an important and entangled cross-talk or feedback between mitochondrial ROS production and p53 activity in cardiac muscle, leading to a global homeostasis or change in cellular activity.

Our data showed that the increase in age decreased mitochondrial Δψ and increased mitochondrial ROS production, accompanied by an increased protein content of p53 in mitochondria of cardiac muscle. The present data suggest indirectly that p53 translocation to mitochondria and its effects on mitochondrial ROS production play an adverse role in the early phase of cardiac aging. Our results provided a new evidence for exercise to prevent cardiac aging that exercise increased mitochondrial Δψ and decreased p53 protein content in mitochondria of old mice, but not in young mice. To testify the role of p53 translocation to mitochondria by which exercise affected COX biogenesis, we found here that PFTμ, an inhibitor blocking p53 translocation to mitochondria, increased COX biogenesis in older mice, but not in young mice. These effects of PFTμ on COX biogenesis only in the older mice made it more convincing that exercise increased mitochondrial function and COX biogenesis via inhibiting p53 translocation to mitochondria.

In summary, we demonstrated that age-related changes were attenuated more or less by exercise in mitochondrial activity, COX biogenesis and p53 activity targeting at SCO2 and mitochondria. The decreased p53 binding to the SCO2 promoter and the increased p53 mRNA and its translocation to mitochondria during the early phase of aging, were attenuated or reversed clearly in older mice by exercise. This study provided a correlative evidence that endurance exercise was able to prolong the equilibrium state of p53 expression and distribution relative to age-related changes, thereby delaying mitochondrial COX decay in aging cardiac muscle.

## Materials and Methods

Upon approval by the local government, all experiments were performed in accordance with the guidelines for the use of laboratory animals published by China Ministry of Health (No.55 order, ordained on 25 Jan, 1998). All experimental procedures were approved by the Experimental Animal Care and Use Committee at East China Normal University (ECNU 2006-05).

### 1. Animals

Male ICR/CD-1 mice were purchased from Sino-British Sippr/BK Lab Animal Ltd., CO (Shanghai, China) at 4∼5 weeks of age or 8∼9 months of age, and were housed at East China Normal University until sacrifice at different age (2, 4, 8∼9 months old or the oldest), respectively. The oldest mice derived from the animals at 8∼9 months old and were fed until the natural mortatity was more than 50%, finally the number of the oldest group was 9. The second groups of male ICR/CD-1 mice at 1 or 8∼9 months of age were also purchased, one week after arriving at our facility, mice were randomly assigned to one of four groups: (1) young sedentary (YS), (2) old sedentary (OS), (3) young with treadmill running (YEx) and (4) old with treadmill running (OEx). A performance test to determine the maximum running capacity for each mouse was performed firstly by running the mice for 5 min at 13 m/min and then increasing the speed 1 m/min per minute until exhaustion. The young mice's maximum capacity was determined averagely to be 42 m/min, and the old mice's maximum capacity was 33 m/min. YEx and OEx mice were accustomed to a 4-week treadmill running at 65% of that speed at which the mice reached exhaustion for 1 h, 6 days/wk. Thus, the young mice were run at 28 m/min, and the old mice were run at 22 m/min. All animals were housed in a temperature (21±2°C) and light-controlled (12∶12 h light-dark cycle) environment, and were provided diets and water ad libitum. Body weights of all mice were recorded weekly.

### 2. Pifithrin-μ administration

Pifithrin-μ (PFTμ, Sigma, CAS#: 64984-31-2), an inhibitor of p53 binding and anti-apoptotic, which directly inhibits p53 binding to mitochondria, was dissolved in DMSO and saline. The mice (young and older) received intraperitoneal injection of pifithrin-μ (3 mg/kg body weight/2 day) for 3 weeks. As a control, the vehicle (0.5% DMSO in saline) was injected to the control mice.

### 3. Tissues extraction and mitochondrial isolation

Upon sacrifice, cardiac muscle was removed, rinsed in PBS solution, dried with filter paper, a part of fresh tissue was used for mitochondrial isolation. The rest was frozen in liquid nitrogen. Mitochondria were prepared using differential centrifugation as described previously [61]. For mitochondrial isolation, fresh tissues were rinsed with PBS and put into ice-cold isolation buffer (0.075 M sucrose, 0.225 M sorbitol, 1 mM EGTA, 0.1% fatty acid-free bovine serum albumin (BSA), and 10 mM Tris–HCl, pH 7.4). Tissues were sheared carefully to mince, rinsed to get rid of residual blood, and then homogenized in 1 ml isolation buffer per 100 mg tissue. The homogenate was centrifuged at 1000 g for 5 min at 4°C using a Beckman centrifuge (Avanti J-26XP); the resulting supernatant was decanted and saved. The pellet was washed once with isolation buffer. The supernatant were combined and centrifuged at 9,000 g for 10 min at 4°C. The mitochondrial pellet was washed and centrifuged twice at 15,000 g for 2 min at 4°C with isolation buffer. Mitochondrial protein content was assayed using BSA as a standard according to Bradford.

### 4. Assays of mitochondrial function and oxidative stress

The dichlorofluorescin diacetate (H_2_-DCFDA) was used as a probe to detect mitochondrial ROS generation as described previously [62]. JC-1, the fluorescent, lipophilic and cationic probe, was employed to measure the mitochondrial membrane potential (Δψ) according to the manufacturer's directions as described previously [63]. ATP synthase activity was measured in isolated and freeze-thawed mitochondria as oligomycin-sensitive ATPase activity using an assay coupled with pyruvate kinase which converts the ADP to ATP and produces pyruvate from phosphoenolpyruvate. To assay lipid peroxidation (LPO), MDA derived from polyunsaturated fatty acid peroxides was evaluated by MDA assay kit (Nanjing Jiancheng Biotech, Nanjing, China). GSH/GSSG ratio and Mn-SOD activity was measured by colorimetric analysis with the Assay Kits (Nanjing Jiancheng Biotech, Nanjing, China).

### 5. Quantitative real-time PCR(qPCR) and mtDNA content assay

Total RNA was prepared from ∼100 mg of frozen tissues using TRIzol (Invitrogen, Chromos, Singapore) and purified according to the instructions included. Double-stranded cDNA was synthesized from ∼1 µg of total RNA using ReverTra Ace® qPCR RT Kit (TOYOBO, Osaka, Japan). Real-time PCR reactions were set up using the SYBR-Green PCR kit (TOYOBO, Osaka, Japan) and were cycled in StepOne™ Real-Time PCR System (Applied Biosystems, CA, USA). In brief, RNA concentration were estimated by measuring the absorbance at 260 nm, and purity was assessed by 260 nm/280 nm absorbance ratio. Total RNA was denatured at 65°C for 5 min, cooled immediately on ice, and reverse transcribed. The reaction was assessed at 37°C for 15 min and at 98°C for 5 min. PCR was performed in a fluorescence temperature cycler containing 4 pmol of each primer, 2.0× Master SYBR Green I (contains Taq DNA polymerase, reaction buffer, dNTP mix, SYBR Green I dye, and 10 mM MgCl_2_), and 2.0 µl template in a total volume of 20 µl. The amplification occurred in a three-step cycle (denaturation at 95°C for 15 s, annealing at 61°C for 30 s, extension and data collection at 72°C for 45 s) for 40 cycles. The fluorescence signal was plotted against cycle number for all samples and external standards. The abundance of target mRNA was normalized to that of β-actin.

Mitochondrial DNA content was also determined by qPCR. Briefly, total DNA was extracted and purified from cardiac muscle. 10 nanograms of DNA was used to quantify mitochondrial and nuclear DNA markers. β-actin was also used as a nuclear DNA marker. AK140265 and Cytb were used as mtDNA markers. PCR was performed as stated above. Primer pairs were designed based on GenBank reference sequences and listed in Table S1.

### 6. Western blotting analysis

For assays of p53 protein level in isolated mitochondria, equal amounts of mitochondrial protein were run on 10% SDS- polyacrylamide (120V; Bio-Rad, Hercules, CA). After electrophoretic separation, proteins were transferred (1 h, Criterion blotter; Bio-Rad) to PVDF membranes. After Ponceau S staining and destaining, membranes were blocked in 5% nonfat dry milk powder (Shanghai Sangon, China) in Tris-buffered saline containing 0.1% Tween 20 (TBST) for 1 h at room temperature. Thereafter, a dilution of the primary specific antibody in 5% TBST was added and incubated overnight at 4°C on a shaker. After the membranes were washed 3 times for 10, 10, 10 min in 5% TBST, the membranes were incubated with a 1∶2,000 dilution of the horseradish peroxidase-conjugated secondary antibody in 5% TBST for 1 h at room temperature. Thereafter, the membranes were washed 3 times in TBST for 10, 10, 10 min [64]. Finally, the BeyoECL Plus chemiluminescent/fluorescent substrate (Beyotime, China) was applied, and the chemiluminescent signal was captured with an Alpha Innotech Fluorchem SP imager (FluorChem FC2, San Leandro, CA). The digital images were analyzed using Alpha Innotech software. Density of the target band was normalized and expressed in arbitrary optical density units. Western blot analysis was performed using the following antibodies and dilutions: p53 (sc-71819, Santa Cruz Biotechnology, 1∶200), COXIV (sc-58348, Santa Cruz Biotechnology, 1∶200).

### 7. Chromatin immunoprecipitation (ChIP) assay

ChIP assay was performed using a kit from Upstate Cell Signaling Solutions as described previously [65]. In brief, diced tissues (∼100 mg) was cross-linked in DMEM containing 1% formaldehyde for 10 min at 37°C and lysed on ice in 500 µl SDS lysis buffer (1% SDS, 10 mM EDTA, 50 mM Tris, 0.5 mM PMSF, and protease inhibitors). Chromatin was sheared to fragments by 8∼10×15 s bursts of sonication. Following centrifugation (13000 g for 10 min at 4°C), 100 µl of supernatant, containing chromatin fragments, were diluted 10-fold in a buffer (0.01% SDS, 1.1% Triton X-100, 1.2 mM EDTA, 16.7 mM TrisHCl, and 167 mM NaCl), precleared with salmon sperm DNA/protein A agarose (Upstate #16-157), and centrifuged again (1000 g for 1 min at 4°C). The resultant supernatant, referred to as input sample, was immunoprecipitated with p53 antibody and protein A agarose. Following centrifugation, the protein A agarose/antibody/histone complex was washed for 3–5 minutes on a rotating platform with 1 ml of each of the buffers listed in the order as given below: Low Salt Immune Complex Wash Buffer; High Salt Immune Complex Wash Buffer; LiCl Immune Complex Wash Buffer; 1×TE (two washes). The pellet were eluted in a buffer consisting of 1% SDS and 0.1 M NaHCO_3_ and reverse cross-linked by adding 5 M NaCl followed by incubation at 65°C for 4 h. The coimmunoprecipitated DNA was purified by phenol- chloroform extraction and resuspended in 20 µl H_2_O. A 208 bp fragment corresponding to nucleotides 127 to 334 of the mouse SCO2 promoter, containing the p53 binding site, were amplified and quantified by qPCR as described above using the following primers (+ve primers): 5-GAGCAGCTCCTCTTGCTCTC-3 (forward); 5-TCTTTGGATTAGCGGACAGG-3 (reverse). A pair of primers (-ve primers) specific to a region ∼1.6 kb downstream from the SCO2 start site was used as a negative control for nonspecific binding of chromatin to the immuno-precipitation antibody: 5-CTCATCGGGGCAAATATCAG-3 (forward); and 5-AGGGCTGAGAAGGAACA GTG-3 (reverse). Purified DNA from input sample that did not undergo immuno- precipitation was PCR amplified and used to normalize signals from ChIP assays. The DNA content in these control reactions was 1% of those used in parallel immuno-precipitation reactions. A PCR reaction using equal genomic DNA was also run with each set of PCR reactions to allow comparison between different experiments.

### 8. Statistical analysis

All data are reported as mean ± SEM. Statistical analysis was performed using SPSS 15.0 software. Statistical differences between treatments were determined using a one-way ANOVA or a Student's t-test as appropriate. For all tests the significance level was set at P<0.05.

## Supporting Information

Figure S1 **Scheme of mouse chr15:89202068-89204249.** Description: Mus musculus SCO cytochrome oxidase deficient homolog 2 (yeast) (Sco2), nuclear gene encoding mitochondrial protein. Entrez Gene: 100126824 PubMed on Product: protein SCO2 homolog mitochondrial precursor Stanford SOURCE: NM_001111288.
(TIF)

Table S1 **Gene primers sequences for real-time PCR (5′ to 3′).**
(DOC)

Table S2 **The cycle threshold values (Ct) of all samples displayed on the PCR System in the ChIP assays.** Cross-linked chromatin was sheared and immunoprecipitated with nonspecific antibody and anti-p53 antibody. After reversal of cross-links, immunoprecipitated DNA fragments, input DNA sample, no-template control (NTC) were all detected by real-time PCR using primers (+ve: flanking the p53 sites in the mouse SCO2 promoter; -ve: flanking a region ∼1.6 kb downstream from the SCO2 start site). The cycle threshold values (Ct) those displayed on the PCR System are presented as mean ± SEM (n = 28) or “Undetermined” in the Table. “Undetermined” means that the Ct value is much more than 40 cycles, and that the targeted DNA fragments is very little. All these data confirmed the specificity of the ChIP assays.
(DOC)
