# Supplementary figures and images for: Physical Exercise Regulates p53 Activity Targeting SCO2 and Increases Mitochondrial COX Biogenesis in Cardiac Muscle with Age
Source: PLoS One. 2011 Jul 7;6(7):e21140. doi: 10.1371/journal.pone.0021140 (PMC3131270; doi:10.1371/journal.pone.0021140)

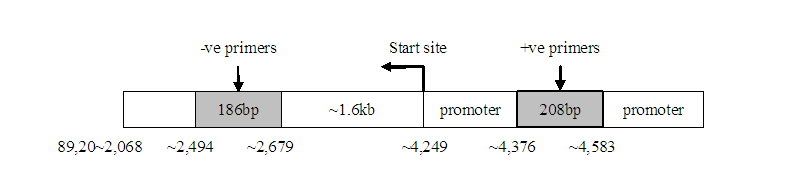

Supplement: Figure S1 — Scheme of mouse chr15:89202068-89204249. Description: Mus musculus SCO cytochrome oxidase deficient homolog 2 (yeast) (Sco2), nuclear gene encoding mitochondrial protein. Entrez Gene: 100126824 PubMed on Product: protein SCO2 homolog mitochondrial precursor Stanford SOURCE: NM_001111288. (TIF) [file pone.0021140.s001.tif]
