# Supplementary material for: Physical Exercise Regulates p53 Activity Targeting SCO2 and Increases Mitochondrial COX Biogenesis in Cardiac Muscle with Age
Source: PLoS One. 2011 Jul 7;6(7):e21140. doi: 10.1371/journal.pone.0021140 (PMC3131270; doi:10.1371/journal.pone.0021140)
Supplement: Table S1 — Gene primers sequences for real-time PCR (5′ to 3′). (DOC) [file pone.0021140.s002.doc]

**Table S1. Gene primers sequences for real-time PCR (5’ to 3’)**

| Gene | Forward Primer | Reverse Primer |
| --- | --- | --- |
| AK140265 | CGATAAACCCCGCTCTACCT | AGCCCATTTCTTCCCATTTC |
| Cytb | AACATACGAAAAACACACCCATT | AGTGTATGGCTAAGAAAAGACCTG |
| COXIV | CGCTGAAGGAGAAGGAGAAG | GCAGTGAAGCCAATGAAGAA |
| COXVb | GGAAGTGCATCTGCTTGTCTC | TAGGGACACCACCTCCAGAA |
| SCO1 | CTGGACTGACCACATCTTTT | CTGGTTTTCAGTGGTAGACC |
| SCO2 | CTCATCGGGGCAAATATCAG | AGGGCTGAGAAGGAACAGTG |
| COXII | ACGAAATCAACAACCCCGTA | CTAGGGAGGGGACTGCTCAT |
| PPARγ | ATGGAAGACCACTCGCATT | AATCCTTGGCCCTCTGAGAT |
| ERRα | CAACCTATGTGGCAGGGTG | TGCATGCAGCACTGTACC |
| PGC-1α | GCATGAGTGTGTGCTGTGTG | TCCAGGAATCATTGCATCTG |
| PGC-1β | TGAGGTGTTCGGTGAGATTG | CCATAGCTCAGGTGGAAGGA |
| Tfam | TGCAGTTTCTTGGTCAGCAT | TGCACAACTGGTAACCATCA |
| TFB1M | AAGTTGATGTAGGAGTGGTG | ATGTCTGCCAACTGTAACAG |
| TFB2M | AGACACACCTATCCTAGGCC | CTCTCTGCCGTAGATCAATG |
| p53 | GTAGGAAGGCGCGTGGTAG | CAGTTACAGGAACCCCGAG |
| β-actin | TGTTACCAACTGGGACGACA | CTATGGGAGAACGGCAGAAG |
