# Supplementary material for: Physical Exercise Regulates p53 Activity Targeting SCO2 and Increases Mitochondrial COX Biogenesis in Cardiac Muscle with Age
Source: PLoS One. 2011 Jul 7;6(7):e21140. doi: 10.1371/journal.pone.0021140 (PMC3131270; doi:10.1371/journal.pone.0021140)
Supplement: Table S2 — The cycle threshold values (Ct) of all samples displayed on the PCR System in the ChIP assays. Cross-linked chromatin was sheared and immunoprecipitated with nonspecific antibody and anti-p53 antibody. After reversal of cross-links, immunoprecipitated DNA fragments, input DNA sample, no-template control (NTC) were all detected by real-time PCR using primers (+ve: flanking the p53 sites in the mouse SCO2 promoter; -ve: flanking a region ∼1.6 kb downstream from the SCO2 start site). The cycle threshold values (Ct) those displayed on the PCR System are presented as mean ± SEM (n = 28) or “Undetermined” in the Table. “Undetermined” means that the Ct value is much more than 40 cycles, and that the targeted DNA fragments is very little. All these data confirmed the specificity of the ChIP assays. (DOC) [file pone.0021140.s003.doc]

**Table S2. The cycle threshold values (Ct) of all samples displayed on the PCR System in the ChIP assays.**

|  | Ct (+ve primer) | Ct (-ve primer) |
| --- | --- | --- |
| NTC | Undetermined | Undetermined |
| IgG:ChIP | Undetermined | Undetermined |
| Input DNA | 30.38±1.10 | 30.29±1.01 |
| p53:ChIP | 31.28±0.20 | Undetermined |
